# An Integrated Approach to Assess Knowledge/Perceptions and Attitudes/Practices (KAP) Regarding Major Neglected Tropical Diseases Endemic in the Mbengwi Health District, North West Region, Cameroon

**DOI:** 10.1007/s44197-021-00010-8

**Published:** 2021-10-26

**Authors:** Laurentine Sumo, Ngum H. Ntonifor, Cédric G. Lenou-Nanga, Nicanor Chenkumo-Kengmoni, Vanessa T. Amana-Bokagne, Chembo G. Awah, Yannick Niamsi-Emalio, Hugues C. Nana-Djeunga

**Affiliations:** 1 Department of Biological Sciences, Faculty of Science, University of Bamenda, Bambili, Cameroon; 2 Centre for Research on Filariasis and other Tropical Diseases (CRFilMT), Yaoundé, Cameroon

**Keywords:** Lymphatic filariasis, Onchocerciasis, Soil transmitted helminthiasis, KAP, Mbengwi health district, Cameroon

## Abstract

**Background and objectives:**

Preventive chemotherapy (PCT) is the main strategy currently used to control and/or eliminate onchocerciasis, lymphatic filariasis (LF) and soil transmitted helminthiasis (STH), and community participation (through implementation or adherence to PCT) is critical. This study aimed at investigating knowledge/perceptions of populations of the Mbengwi health district (North West Region, Cameroon), in relation to their attitudes/practices regarding the most prevalent neglected tropical diseases (NTDs).

**Methods:**

A household-based cross-sectional survey was carried out in the Mbengwi health district (North West Region, Cameroon) using the cluster sampling technique. Clusters were selected using the probability proportionate to estimate size strategy. In each cluster, the random walk technique was used for the selection of households, and a structure questionnaire was administered to 2–3 of its members.

**Results:**

A total of 254 households from 26 clusters were visited, and 514 individuals were interviewed. The sex ratio of interviewees (1.08) was unbiased, and their ages ranged between 10 and 99 years old. Though most of the respondents declared having already heard of these NTDs (41.6%, 73.9% and 90.5% for onchocerciasis, LF and STH, respectively), only a minority of them were aware of how they are acquired/transmitted (8.9%, 9.2% and 32.7% for onchocerciasis, LF and STH, respectively), or prevented (23.1%, 18.9% and 47.2% for onchocerciasis, LF and STH, respectively).

**Conclusions:**

This study revealed poor knowledge/perceptions and wrong attitudes/practices of interviewees as regards to these NTDs, and these misconceptions can seriously affect the adherence and contribution of populations to the success of PCTs. It appears compulsory to reinforce information, education, and communication, with a focus on the rationale and importance behind PCTs, to optimize/improve community participation.

**Supplementary Information:**

The online version contains supplementary material available at 10.1007/s44197-021-00010-8.

## Introduction

Neglected tropical diseases (NTDs) are among the world's most common conditions prevailing in tropical and subtropical settings and disproportionately affect the poorest [1, 2]. Since the update of the NTD portfolio in 2017 by the addition of chromoblastomycosis and other deep mycoses, scabies and other ectoparasites and snakebite envenoming [3], the burden of NTDs is yet to be updated. The previously known 17 NTDs were recognized to affect more than 2 billion people in developing countries in 2013, STH infections (ascariasis, trichuriasis, and ancylostomiasis/necatoriasis) accounting for more than three-quarters of the total NTD infections prevalence. In addition to their morbidity, the number of death attributable to the 17 NTDs prioritized by the WHO plus “other NTDs” in 2013 was estimated to be equivalent to more than half of the malaria deaths and more than double due to tuberculosis [4–6].

Despite their high burdens, these NTDs are preventable and/or treatable. The control and/or elimination of these NTDs are now on top of the agenda of endemic countries’ control programs and stakeholders in their efforts to achieve Millennium Development Goals for sustainable poverty reduction [1, 4, 7]. Five public-health interventions: (1) preventive chemotherapy (PCT), (2) innovative and intensified disease management, (3) vector control and pesticide management, (4) safe drinking-water, basic sanitation and hygiene (WASH) and (5) zoonotic disease management, have been identified to accelerate the prevention, control, elimination and eradication of NTDs, more effective impact being achieved when these interventions are combined [1, 8].

Success in controlling these NTDs have recently been achieved in a number of countries [9], but the trends towards elimination remains unsatisfactory [1]. Indeed, these NTDs are still persisting in most endemic areas, especially when long-term sustainable efforts are required as it is the case of the five major ‘tool-ready’ NTDs (lymphatic filariasis, onchocerciasis, schistosomiasis, soil-transmitted helminthiasis and trachoma). Among the reasons identified to hinder the elimination of these NTDs, sub-optimal treatment coverage and the presence of systematic non-compliers in communities are significant [10]. The implication and contribution/participation of populations in this machinery, through implementation of mass drug administration (MDA) or adherence to PCT, are highly needed and can appear critical in the momentum towards elimination. For instance, it was demonstrated that blackfly biting rates have declined by 89–99% after members of some communities in Northern Uganda have been mobilized to "slash and clear" the breeding sites of the vegetation that represents the primary onchocerciasis vector larvae attachment point [11]. It was argued that water, sanitation, and hygiene (WASH) interventions are effective at reducing STH infections [12], though most of the children interviewed in the framework of a study conducted in Senegal declared that they usually defecate somewhere else, though their communities were well endowed with pit latrines [13]. This means that the appropriation of control measures by the populations is a key point, and this can be made through appropriate education of endemic communities to improve their knowledge regarding these infections.

The objective of this study was to assess knowledge/perceptions of populations, in relation to their attitudes/practices, regarding the most prevalent NTDs in the Mbengwi health district (North West Region, Cameroon).

## Materials and Methods

### Ethical Considerations

Ethical clearance was granted by the Institutional Review Board of the Faculty of Science of the University of Bamenda. The study was conducted according to the guidelines of the Declaration of Helsinki and approved by the Institutional Review Board of the Faculty of Science of the University of Bamenda. After approval of the local administrative and traditional authorities, the objectives and schedules of the study were explained to community leaders and to all eligible individuals selected in the household. Verbal agreements were obtained from those who agree to participate, under the discretion of community leaders and local guides. The approval of parents or legal guardians of minors was necessary before any procedure. An individual code was attributed to each participant for anonymous data analysis.

### Study Area and Populations

Mbengwi (6°01′ N, 10°00′ E) is the capital of the Momo Division (North West Region, Cameroon), situated at about 20 km to the west of Bamenda town, the Regional capital, and at an altitude ranging from 900 to 2000 m above sea level. The hydrographic network is intricate with many streams and springs (the Momo division is mainly irrigated by the Momo River), and the area has a huge hydroelectricity potential afforded by the Abi falls. The climate of the Mbengwi health district is of mountain monsoon equatorial type, with a short dry season running from September to March, and a long rainy season extending from March to September. The annual average rainfall is 2022.3 mm, and the average maximum yearly temperature is 30 °C [14, 15]. This area is characterized with a long valley stretch surrounded by mountains (most of them counting at least 1500 m in height) and lies in the transitional zone between the western grass fields and forest region. It is highly dominated by the savannah vegetation (especially on the hills) which favors animal rearing. The valley is mainly made up of trees (palm trees, raffia palms and many fruit trees), and is favorable for settlement and agriculture. Forest characteristics are highly observed at the western part of the area and favors the cultivation of cash crops like cocoa [14, 15]. The Mbengwi health district is made up of 77 communities/villages organized into 17 health areas. The population size of the health district is about 54,810 inhabitants based on the 2017 health population denominators [16].

### Study Design and Sampling Strategy

This study was designed as a representative 2-staged clustered cross-sectional survey, with a convenient sampling approach based on the random walk door-to-door recruitment strategy as recommended by the World Health Organization. Briefly, clusters (communities) were selected using the probability proportional to estimate size (PPES) strategy [17], and in each selected community, the random walk technique [18] was used for households’ selection. In each selected household, 2–3 individuals, either males or females, aged 10 years and above, who had been living in the selected cluster for at least 5 years, were randomly selected and underwent in-depth interviews. Individuals aged < 10 years were excluded as they were less likely to provide accurate enough answers to such in-depth interviews. Respondents were organized into three different age classes: 10–20 years old, 21–30 years old and 31 years and over (usually the head of household). A structured questionnaire was administered by three trained interviewers, assisted by autochthones speaking the local language (local guides), to collect participants’ socio-demographic and socio-economic characteristics (gender, age, village of residence, profession, educational level, means of locomotion), and to assess whether they have ever heard about the targeted NTDs (onchocerciasis (ICD classification: 1F6A), lymphatic filariasis (ICD classification: 1F66.3) and soil transmitted helminthiasis (ICD classification: 1F62 for ascariasis; 1F6G for trichuriasis and 1F68 for hookworm diseases)), as well as the level of their knowledge/perceptions in relation to their attitudes/practices as regards to the mode of transmission, clinical manifestations and the means to prevent and treat these parasitic diseases.

### Data Analysis

All relevant data were recorded into a purpose-built Microsoft Access database and subsequently transferred to R software, version 3.6.1 (The R Foundation for Statistical Computing) for statistical analysis. Categorical variables (gender, age classes, occupation, urbanization) were summarized using frequencies, and continuous variables (age) were described using median (interquartile range, IQR). To better evaluate respondents’ knowledge/perceptions, attitudes/practices, a score of “1” was attributed to each correct answer and “0” to each wrong answer to the different questions asked. The overall KAP score for a given study participant was the sum of all answers for a specific disease. These overall scores were then categorized, both for knowledge/perceptions and attitudes/practices, as poor or average/good (see the supplementary material Table S1). KAP scores were compared between respondents’ socio-demographic and socio-economic characteristics using the Chi-Square and Fisher exact probability tests. Univariate and multivariate logistic regression models were performed to assess the strength of the association between knowledge/perceptions and attitudes/practices. Odds Ratio (OR) with 95% confidence interval (CI) were computed to test the association between the response variable (KAP score) and the socio-demographic variables (gender, age group, education level, and urbanization level). Non-overlapping 95% CI or *p*-values ≤ 5% were considered as statistically significant.

## Findings

### Socio-Demographic and Socio-Economic Status of Study Participants

Overall, 13 health areas (76.5% coverage) and 26 communities (33.8% coverage) were visited during the survey. Table 1 summarizes socio-demographic and socio-economic characteristics of the survey respondents. A total of 254 households selected in 26 clusters were visited across the Mbengwi health district, and 514 participants were interviewed. The sex ratio (Male/Female) of enrollees was 1.08, their age ranging between 10 and 99 years old (median: 35 years old; IQR: 23–49 years old). The educational level of most of the study participants was primary/secondary (84.1%), only a few being illiterate. More than half (50.4%) of the interviewees were farmers, living almost exclusively in rural (46.5%) and semi-rural/semi-urban (39.3%) settings, most of them using motorbike (58.2%) as means of locomotion.

**Table 1:** Socio-demographic and socio-economic characteristics

Variables	No. individuals (%)
Urbanization level	
Rural	230 (46.5)
Semi urban	202 (40.8)
Urban	63 (12.7)
Occupation	
Farmer	259 (50.4)
Student/Teacher	124 (24.1)
Other^a^	131 (25.5)
Gender	
Female	247 (48)
Male	267 (52)
Age group	
(10–21)	100 (19.5)
(21–31)	126 (24.5)
(31–100)	288 (56)
Educational level	
Illiterate	50 (9.9)
Primary	243 (47.9)
Secondary	189 (37.3)
Higher	25 (4.9)

^a^Drivers, traders, hairdressers, dressmakers

### Awareness of the Targeted NTDs

More than half of the respondents (58.0%) indicated that they have never heard about onchocerciasis (Table 2). For those who were aware of this debilitating disease, the most common information sources were community members (45.8%) and health personnel (40.2%). Regarding LF, 380 (73.9%) respondents indicated that they had already heard about this filarial infection, the most common source of information being community members (Table 2). STH infections were well known to the respondents (90.5%), mostly from health personnel (61.1%) (Table 2).

**Table 2:** Knowledge/perceptions, attitudes/practices of populations with regards to onchocerciasis, lymphatic filariasis and soil transmitted helminthiasis

Indicative questions	Response categories	Onchocerciasis *N* (%)	LF *N* (%)	STH *N* (%)
Have you heard about this NTD?	Yes	212 (41.2)	379 (73.7)	462 (89.9)
	No	300 (58.4)	132 (25.7)	50 (9.7)
	Refuse to answer	2 (0.4)	3 (0.6)	2 (0.4)
If « Yes»				
By which means?	Radio	21 (10.0)	23 (6.1)	5 (1.1)
	Television	5 (2.3)	11 (2.9)	4 (0.9)
	Poster	9 (4.2)	34 (9.0)	2 (0.4)
	Health personnel	86 (41.1)	111 (29.3)	284 (61.5)
	Member of the community	98 (46.2)	220 (58.0)	156 (33.8)
How can you get this disease?	Flies	19 (9.0)	13 (3.4)	NA
	Mosquito	21 (10.0)	35 (9.2)	NA
	Inherited from parent/family	2 (0.9)	1 (0.3)	NA
	Worms enter through the foot	NA	NA	4 (0.9)
	Bad hygiene*	NA	NA	151 (32.7)
	Not washing hands	NA	NA	22 (4.8)
	Inherited from parent/family	NA	NA	1 (0.2)
	Don’t know	145 (68.4)	308 (81.3)	255 (55.2)
What does this disease cause?	Pruritus or itching	0 (0)	NA	NA
	Skin disease	6 (2.8)	NA	NA
	Eye disease	155 (73.1)	NA	NA
	Elephantiasis	NA	328 (86.5)	NA
	Hydrocele	NA	9 (2.4)	NA
	Stomachache	NA	NA	329 (71.2)
	Diarrhea	NA	NA	75 (16.2)
	Vomiting	NA	NA	67 (14.5)
	Don’t know	48 (22.6)	42 (11.1)	42 (9.1)
How can you prevent this disease?	Take medicine	41 (19.3)	57 (15.0)	136 (29.4)
	Kill flies	8 (3.8)	10 (2.6)	NA
	Use a bed net	8 (3.8)	5 (1.3)	NA
	Use latrines	NA	NA	2 (0.4)
	Good Hygiene	NA	NA	80 (17.3)
	Don’t know	146 (68.9)	307 (81.0)	226 (48.9)
What can you do to treat this disease?	Take medicine	91 (42.9)	112 (29.6)	342 (74.0)
	Kill Flies	0 (0)	0 (0)	NA
	Use latrines	NA	NA	2 (0.4)
	Good hygiene	NA	NA	2 (0.4)
	Don’t know	112 (52.8)	234 (61.7)	112 (24.2)

### Knowledge of Modes of Transmission, Clinical Manifestations and Prevention/Treatment Means of Onchocerciasis, Lymphatic Filariasis and Soil Transmitted Helminthiasis

Among those who were aware of these diseases, only few of them (8.9%) identified blackflies as the river blindness transmission agent (Table 2). A similar situation was observed for LF, less than quarter (10.0%) of the respondents indicating that mosquito act as vector (Table 2). Likewise awareness, respondents’ knowledge regarding STH transmission was higher as compared to onchocerciasis and LF, though remaining low (Table 2).

Of the respondents who were aware of onchocerciasis, an important proportion of study participants (72.4%) identified eye lesions among clinical signs (Table 2). Elephantiasis of lower limb was reported by 86.3% of those who were aware of LF, while only 2.4% of them identified hydroceles as one of the LF clinical sign (Table 2). Stomach ache (70.8%), diarrhea (16.1%) and vomiting (14.4%) were the main clinical manifestations reported by study participants who were aware of STH infections (Table 2).

Of those respondents who were aware of onchocerciasis, prevention and treatment approaches were not known by more than half of the respondents. Among those participants who knew that medicines can be used for chemoprevention (19.2%) or chemotherapy (42.5%), only few of them knew that ivermectin was the drug routinely used (Table 2).

### KAP scores of Onchocerciasis, Lymphatic Filariasis and Soil Transmitted Helminthiasis

Different variables of knowledge/perception and attitudes/practices of the targeted NTDs were categorized using a scoring approach (Supplementary material Table S1). The average knowledge score regarding onchocerciasis was very low (0.84); almost 75% of respondents had a knowledge score less than or equal to 1, for a maximum score equal to 4. The knowledge score for LF was similar to that of onchocerciasis. Indeed, of the three correct answers expected (maximum score equal to three as shown in the Table S1), 76.3% provided only one correct answer (Supplementary material Table S2) and none provided three correct answers. Regarding STH knowledge, out of the five correct answers expected (maximum score equal to three as shown in the Table S1), 50% of the respondents provided only one or two correct answers. The average STH knowledge score was 1.33 (maximum score equal to six as shown in the Table S1).

Regarding attitudes and practices, 15.0%, 7.9% and 35.3% of respondents had a score of 2 for onchocerciasis, LF and STH, respectively (maximum score equal to three as shown in the Table S1). The proportions of interviewees decreased when scores of both knowledge/perception and attitudes/practices were increasing (Supplementary material Table S2) for the three diseases. The categorization of these scores into poor or good knowledge/perceptions and attitudes/practices is summarized in Supplementary material Table S3. The proportion of individuals exhibiting good knowledge/perceptions does not exceed 11.0% for each disease. The proportions of interviewees with good attitudes/practices were 37.4%, 16.4 and 8.7% for STH, onchocerciasis and LF, respectively (Supplementary material Table S3).

### Association Between KAP Scores of Targeted NTDs and Different Covariates

The univariate analysis revealed that most of the socio-demographic characteristics are associated neither with the knowledge/perceptions, nor with the attitudes/practices of these targeted diseases (Table 3). However, the elder people (aged > 21 years) had poorer knowledge/perceptions of onchocerciasis than the youngest, whereas knowledge/perceptions and attitudes/practices regarding STH was found to be increasing with age of enrollees. Also, interviewees living in urban areas exhibited poorer knowledge/perceptions and attitudes/practices with regard to LF, compared to their counterparts living in rural settings (Table 3).

**Table 3:** Crude Odds Ratios of KAP variables related to onchocerciasis, lymphatic filariasis and soil transmitted helminthiasis according to gender, age education and urbanization

Variables	Onchocerciasis		Lymphatic filariasis		STH	
	Knowledge/Perceptions	Attitudes/Practices	Knowledge/Perceptions	Attitudes/Practices	Knowledge/Perceptions	Attitudes/Practices
Gender						
Female	1	1	1	1	1	1
Male	1.05^NS^	1.00^NS^	0.98^NS^	0.98^NS^	1.01^NS^	1.02^NS^
Age groups						
(10–21)	1	1	1	1	1	1
(21–31)	0.84**	1.12^NS^	0.97^NS^	0.98^NS^	1.01^NS^	1.11^NS^
(31–100)	0.92*	1.09^NS^	0.99^NS^	0.98^NS^	1.09**	1.18***
Educational level						
Illiterate	1	1	1	1	1	1
Primary	0.44^NS^	1.09^NS^	1.03^NS^	1.02^NS^	1.05^NS^	1.12^NS^
Secondary	0.17^NS^	1.09^NS^	1.05^NS^	1.03^NS^	1.04^NS^	1.14^NS^
University	0.18^NS^	1.30*	1.19**	1.03^NS^	1.02^NS^	1.19^NS^
Urbanization level						
Rural	1	1	1	1	1	1
Semi urban	1.01^NS^	1.04^NS^	1.01^NS^	1.03^NS^	0.97^NS^	1.00^NS^
Urban	0.95^NS^	1.00^NS^	0.93^NS^	0.92**	0.92*	0.92^NS^

*p < 0.1

**p < 0.05

***p < 0.01

*NS* not significant

### Relationship Between Knowledge/perceptions of Respondents and Their Attitudes/practices

The association between knowledge/perceptions and attitudes/practices of interviewees is shown in Table 4. Compared to individuals exhibiting poor knowledge/perceptions of the targeted NTDs, those with average/good knowledge/perceptions exhibited better attitudes/practices towards onchocerciasis (aOR = 1.33; *p* = 0.003), LF (aOR = 1.32; *p* < 0.001) and STH (aOR = 1.41; *p* < 0.001). Regarding the other co-variables included in the logistic regression model (gender, age group, education level, and urbanization level), only age groups (≥ 31 years) and educational level (higher than secondary education level) were significantly associated with the attitudes/practices regarding STH (*p* < 0.012).

**Table 4:** Factors associated with knowledge/perceptions of interviewees in the multivariable logistic regression model

	Attitudes/practices onchocerciasis		Attitudes/practices LF		Attitudes/practices STH	
	aOR (95% CI)	*p*	aOR (95% CI)	*p*	aOR (95% CI)	*p*
Knowledge/perceptions						
Poor	1	–	1	–	1	–
Average/good	1.33 (1.11–1.61)	0.003	1.32 (1.21–1.44)	< 0.001	1.41 (1.23–1.27)	< 0.001
Gender						
Female	1		1	–	1	
Male	0.96 (0.87–1.07)	0.489	0.97 (0.92–1.03)	0.375	0.97 (0.89–1.06)	0.555
Age groups						
(10–21)	1		1	–	1	
(21–31)	1.16 (0.98–1.37)	0.078	1.00 (0.91–1.09)	0.991	1.12 (1.08–1.35)	0.086
(31–100)	1.15 (0.99–1.34)	0.071	0.99 (0.91–1.08)	0.817	1.22 (1.08–1.39)	0.002
Educational level						
Illiterate	1		1	–	1	
Primary	1.11 (0.90–1.37)	0.315	1.03 (0.93–1.03)	0.600	1.14 (0.98–1.35)	0.086
Secondary	1.12 (0.90–1.40)	0.310	1.03 (0.92–1.15)	0.666	1.25 (1.05–1.48)	0.012
University	1.29 (0.97–1.71)	0.082	1.00 (0.86–1.17)	0.991	1.27 (0.99–1.64)	0.057
Urbanization level						
Rural	1		1	–	1	
Semi urban	1.07 (0.96–1.19)	0.213	1.03 (0.97–1.10)	0.298	1.03 (0.94–1.13)	0.524
Urban	1.02 (0.87–1.19)	0.832	0.93 (0.86–1.02)	0.123	0.94 (0.82–1.08)	0.381

*CI* confidence interval, *LF* lymphatic filariasis, *STH* soil-transmitted helminthiasis, *aOR* adjusted Odds ratio

## Discussion

The purpose of this study was to assess, through an integrated approach, the knowledge/perceptions of populations, in relation to their attitudes/practices, regarding the most prevalent NTDs in the Mbengwi health district (North West Region, Cameroon).

The awareness of study participants was quite variable from one NTD to another. Indeed, more than half of the study participants declared that they had never heard about onchocerciasis, though the fight against this disease is ongoing and already implemented in this health district for more than 15 years. These findings are contrasting to those collected in Ethiopia [19] where the majority of the study participants were familiar with onchocerciasis; this might be explained by the fact that this filarial disease is not as severe in the North West Region (3.5% in 2011) [20] as in Ethiopia where the endemicity of the disease was high, ranging from 6.9% in the Quara District in northwest Ethiopia, to 85.3% in Teppi in South western [21]. Unlike onchocerciasis, the majority of participants (73.9%) had already heard about LF, from a community member for most of them. This can be explained by the fact that the disease is commonly called elephantiasis, in reference to its most important and visible clinical manifestation. However, these findings are not in line with those collected in Nigeria which revealed that although the region was endemic to LF, the majority of participants (82.1%) were not aware of the disease [22]. Likewise lymphatic filariasis, most of the respondents (90.5%) were well aware of STH infections, mostly from health personnel (61.1%). Indeed, STH are the most widespread NTD over the world, with more than 1.5 billion people infected (~ 1/4 of the world population). These results corroborate those by Yusof et al. [23] and Nath et al. [24] who reported high awareness of STH (62.5%), health workers being the main source of information.

The knowledge of routes of transmission was in general poor, regardless of the targeted disease. Indeed, among those who have ever heard about onchocerciasis, 90.1% did not know that the vector is blackfly, likely due to the low abundance of this vector in the study area. These results are not in line with the findings by Yirga et al. [25] in a study conducted in Southwest Ethiopia where the majority of the respondents associated the disease to the bite of blackflies. Likewise onchocerciasis, the majority of participants did not know that mosquito is the vector of lymphatic filariasis, as was also previously observed elsewhere [26, 27]. Regarding STH infections, 67.3% of participants did not know how this NTD is transmitted, contrarily to the finding by Nath et al. [24] where school-aged children were aware that STH are transmitted through contaminated soil (58.1%) and unhygienic practices (55.7%). In Cameroon, the Schistosomiasis and Soil Transmitted Helminthiasis National Control program is based on the deworming of school-aged children by their teachers. Thus, during the treatment campaigns in primary schools, the out-target population (included in this study) is not educated about the disease and this might explain why study participants were not familiar with the modes of transmission of STH.

Regarding knowledge of clinical manifestations, most of the study participants (72.4%) identified eye disease as a clinical manifestation of onchocerciasis, probably in reference to the common name of this filarial disease, river blindness. Such high level of clinical manifestation knowledge has been observed in a study by Weldegebreal et al. [19], the most reported clinical sign being however itching. A similar trend was observed with lymphatic filariasis, 87.0% of respondents reporting elephantiasis as the main clinical manifestation. These results corroborate those by Amaechi et al. [22] who reported swellings (84.6%) as the most common responses symptoms in Omi irrigation community at North Central of Nigeria. Also, most of the study participants identified stomach ache and diarrhea (87.4%) as the main STH clinical manifestations, as was previously reported in a study conducted in two rural communities of western Ivory Coast [28]. In general, the knowledge of populations as regards with clinical signs of any of the targeted NTDs was high, more likely because these diseases are usually assimilated to their most prominent symptoms.

The majority of study participants (68.2%) was not aware of onchocerciasis prevention means, and almost half of them (52.3%) ignored how to treat this filarial disease. This was quite surprising, especially because the fight against this disease through the community-directed treatment with ivermectin (CDTI) is implemented in this health district since more than 15 years, with acceptable therapeutic coverage. It is worth to, however, mention that CDTI is critically deviating from its initial pathway, with absence of or poor health education at the community level, reduced commitment of community drug distributors (CDDs) likely due to fatigue (treatment ongoing since almost two decades with no clear date for stopping MDA), poor training and supervision, and no more self-monitoring by communities. This might likely explain why populations can adhere to treatment without knowing onchocerciasis prevention and control measures. These results are not in line with those of Weldegebreal et al. [19] in Nigeria where almost all (93.3%) the respondents believed that onchocerciasis is preventable, and 88.4% of them knew that ivermectin is the drug of choice. Regarding lymphatic filariasis, a very little proportion knew LF prevention and treatment strategies, contrarily to what was previously reported in the Omi community in Nigeria, where 61.8% knew how to prevent lymphatic filariasis, and 49.8% reported the use of anti-filarial drug [19]. This might be explained by the deviation observed in CDTI implementation, onchocerciasis and lymphatic filariasis being implemented following the same approach and by the same actors. About one-third of the study participants knew how to prevent STH infections, and 70.1% of them knew that this disease can be treated using drugs, though almost none knew exactly which drug is usually used. These findings were in line with those by Nath et al. [24] in Bangladesh where 64.4% of school-aged children interviewed declared preventing STH by washing hands after defecation, and 75.6% of them knew that to control STH they should take drugs.

Univariate logistic regression revealed that knowledge/perceptions of these three targeted diseases was found to be increasing with age of enrollees, and people living in urban areas exhibited poorer knowledge/perceptions and attitudes/practices compared to their counterparts living in rural settings. Indeed, it is clearly documented that these NTDs are highly endemic and more severe in rural/hard to reach settings, and people are therefore more likely to present better knowledge/perceptions and attitudes/practices compared to their counterparts living in urban settings. Similarly, as the control of these NTDs is taking place on a regular basis (yearly in Cameroon), elder people are also more likely exposed to the communication around. Though these factors have also been observed in previous onchocerciasis, lymphatic filariasis and STH related studies [22, 29], other determinants such as ethnicity, not necessarily evaluated in the framework of this study, might also be associated with of knowledge of populations [19].

Finally, a positive association was found between knowledge/perceptions of respondents and their attitudes/practices with regard to the three targeted NTDs, suggesting that the better populations are educated, the most they can be aware of is the importance of control measures and thus better comply [30].

## Conclusion

This study revealed that study populations of the Mbengwi health district exhibit poor knowledge/perceptions and attitudes/practices as regards to these three PCT-based highly prevalent targeted diseases, although they are endemic in the study area and MDA administered yearly since almost two decades. Misconceptions can seriously affect the adherence and contribution of populations to the success of PCTs, and consequently elimination of the targeted NTDs. Since lack/poor knowledge and wrong beliefs could lead to inappropriate control measures, it appears compulsory to improve education, information and communication (EIC) activities in the study area, with a focus on the rationale and importance behind MDA, to optimize their attitudes/practices, especially community participation to PCTs.

## Supplementary Information

Below is the link to the electronic supplementary material.

Supplementary file1 (DOCX 17 kb)

Supplementary file2 (DOCX 18 kb)

Supplementary file3 (DOCX 18 kb)

## Data Availability

The data presented in this study are available in the article and/or its supplementary materials.

## Abbreviations

CDTI: Community-directed treatment with ivermectin
CI: Confidence interval
CMFL: Community microfilarial load
MDA: Mass drug administration
mf/ss: Microfilariae per skin snip
OR: Odds ratio
PCT: Preventive chemotherapy
SD: Standard deviation
