# Supplementary material for: An Integrated Approach to Assess Knowledge/Perceptions and Attitudes/Practices (KAP) Regarding Major Neglected Tropical Diseases Endemic in the Mbengwi Health District, North West Region, Cameroon
Source: J Epidemiol Glob Health. 2021 Oct 26;11(4):426–34. doi: 10.1007/s44197-021-00010-8 (PMC8664336; doi:10.1007/s44197-021-00010-8)
Supplement: Supplementary file 1 — Supplementary file1 (DOCX 17 kb) [file 44197_2021_10_MOESM1_ESM.docx]

**Supplementary Table S1.** Categorization for KAP scores

| Total number of good answers | Categories | |
| --- | --- | --- |
|  | Poor | Average and good |
| 3 | 0 – 1 | 2 – 3 |
| 4 | 0 – 1 | 2 – 4 |
| 5 | 0 – 2 | 3 – 5 |
| 6 | 0 – 2 | 4 – 6 |
