# Supplementary material for: An Integrated Approach to Assess Knowledge/Perceptions and Attitudes/Practices (KAP) Regarding Major Neglected Tropical Diseases Endemic in the Mbengwi Health District, North West Region, Cameroon
Source: J Epidemiol Glob Health. 2021 Oct 26;11(4):426–34. doi: 10.1007/s44197-021-00010-8 (PMC8664336; doi:10.1007/s44197-021-00010-8)
Supplement: Supplementary file 2 — Supplementary file2 (DOCX 18 kb) [file 44197_2021_10_MOESM2_ESM.docx]

**Supplementary Table S2.** Proportions of interviewees according to their KAP scores for onchocerciasis, lymphatic filariasis and soil transmitted helminthiasis

| Number of good answers | 0 | 1 | 2 | 3 | 4 | 5 | 6 |
| --- | --- | --- | --- | --- | --- | --- | --- |
| Onchocerciasis |  |  |  |  |  |  |  |
| Knowledge/Perceptions n (%) | 53 (24.8) | 144 (67.3) | 15 (7.0) | 2 (0.9) | 0 (0) | - | - |
| Attitudes/Practices n (%) | 112 (52.3) | 67 (31.3) | 32 (15.0) | 3 (1.4) | - | - | - |
| Lymphatic Filariasis |  |  |  |  |  |  |  |
| Knowledge/Perceptions n (%) | 49 (12.9) | 290 (76.3) | 41 (10.8) | 0 (0) | - | - | - |
| Attitudes/Practices n (%) | 232 (61.1) | 115 (30.3) | 30 (7.9) | 3 (0.8) | 0 (0) | - | - |
| Soil Transmitted Helminthiasis |  |  |  |  |  |  |  |
| Knowledge/Perceptions n (%) | 72 (15.5) | 226 (48.6) | 117 (25.2) | 44 (9.5) | 5 (1.1) | 1 (0.2) | 0 (0) |
| Attitudes/Practices n (%) | 112 (24.1) | 179 (38.5) | 164 (35.3) | 10 (2.2) | 0 (0) | - | - |
