# Supplementary material for: An Integrated Approach to Assess Knowledge/Perceptions and Attitudes/Practices (KAP) Regarding Major Neglected Tropical Diseases Endemic in the Mbengwi Health District, North West Region, Cameroon
Source: J Epidemiol Glob Health. 2021 Oct 26;11(4):426–34. doi: 10.1007/s44197-021-00010-8 (PMC8664336; doi:10.1007/s44197-021-00010-8)
Supplement: Supplementary file 3 — Supplementary file3 (DOCX 18 kb) [file 44197_2021_10_MOESM3_ESM.docx]

**Supplementary Table S3.** Proportions of interviewees according to their KAP score categories for onchocerciasis, lymphatic filariasis and soil transmitted helminthiasis

| Variables | Poor | Average - Good |
| --- | --- | --- |
| Onchocerciasis (N=214) |  |  |
| Knowledge/Perceptions n (%) | 197  (92.1) | 17  (7.9) |
| Attitudes/Practices n (%) | 179  (83.6) | 35  (16.4) |
| Lymphatic Filariasis (N=380) |  |  |
| Knowledge/Perceptions n (%) | 339  (89.2) | 41  (10.8) |
| Attitudes/Practices n (%) | 347  (91.3) | 33  (8.7) |
| Soil Transmitted Helminthiasis (N=465) |  |  |
| Knowledge/Perceptions n (%) | 415  (89.2) | 50  (10.8) |
| Attitudes/Practices n (%) | 291  (62.6) | 174  (37.4) |
